# Mitochondrial ROS Triggers KIN Pathogenesis in FAN1-Deficient Kidneys

**DOI:** 10.3390/antiox12040900

**Published:** 2023-04-08

**Authors:** Merlin Airik, Haley Arbore, Elizabeth Childs, Amy B. Huynh, Yu Leng Phua, Chi Wei Chen, Katherine Aird, Sivakama Bharathi, Bob Zhang, Peter Conlon, Stanislav Kmoch, Kendrah Kidd, Anthony J. Bleyer, Jerry Vockley, Eric Goetzman, Peter Wipf, Rannar Airik

**Affiliations:** 1 Division of Nephrology, Department of Pediatrics, UPMC Children’s Hospital of Pittsburgh, Pittsburgh, PA 15224, USA; 2 Department of Genetics and Genomic Sciences, Icahn School of Medicine at Mount Sinai, New York, NY 10029, USA; 3 Department of Pharmacology & Chemical Biology and UPMC Hillman Cancer Center, University of Pittsburgh, Pittsburgh, PA 15213, USA; 4 Division of Genetic and Genomic Medicine, Department of Pediatrics, University of Pittsburgh School of Medicine and UPMC Children’s Hospital of Pittsburgh, Pittsburgh, PA 15224, USA; 5 Nephrology Department, Beaumont Hospital, D09 V2N0 Dublin, Ireland; 6 Department of Paediatrics and Inherited Metabolic Disorders, First Faculty of Medicine, Charles University, 128 08 Prague, Czech Republic; 7 Wake Forest School of Medicine, Winston-Salem, NC 27157, USA; 8 Department of Chemistry, University of Pittsburgh, Pittsburgh, PA 15260, USA; 9 Department of Developmental Biology, University of Pittsburgh, Pittsburgh, PA 15224, USA

**Keywords:** FAN1, karyomegalic interstitial nephritis, DNA damage, oxidative stress, chronic kidney disease

## Abstract

Karyomegalic interstitial nephritis (KIN) is a genetic adult-onset chronic kidney disease (CKD) characterized by genomic instability and mitotic abnormalities in the tubular epithelial cells. KIN is caused by recessive mutations in the FAN1 DNA repair enzyme. However, the endogenous source of DNA damage in FAN1/KIN kidneys has not been identified. Here we show, using FAN1-deficient human renal tubular epithelial cells (hRTECs) and FAN1-null mice as a model of KIN, that FAN1 kidney pathophysiology is triggered by hypersensitivity to endogenous reactive oxygen species (ROS), which cause chronic oxidative and double-strand DNA damage in the kidney tubular epithelial cells, accompanied by an intrinsic failure to repair DNA damage. Furthermore, persistent oxidative stress in FAN1-deficient RTECs and FAN1 kidneys caused mitochondrial deficiencies in oxidative phosphorylation and fatty acid oxidation. The administration of subclinical, low-dose cisplatin increased oxidative stress and aggravated mitochondrial dysfunction in FAN1-deficient kidneys, thereby exacerbating KIN pathophysiology. In contrast, treatment of FAN1 mice with a mitochondria-targeted ROS scavenger, JP4-039, attenuated oxidative stress and accumulation of DNA damage, mitigated tubular injury, and preserved kidney function in cisplatin-treated FAN1-null mice, demonstrating that endogenous oxygen stress is an important source of DNA damage in FAN1-deficient kidneys and a driver of KIN pathogenesis. Our findings indicate that therapeutic modulation of kidney oxidative stress may be a promising avenue to mitigate FAN1/KIN kidney pathophysiology and disease progression in patients.

## 1. Introduction

Bi-allelic pathogenic variants in the FAN1 gene lead to a multisystem disorder that includes chronic kidney disease, pulmonary infections and fibrosis, and increased susceptibility to cancer [1,2]. The major clinical manifestation of this condition is chronic tubulointerstitial kidney disease, with slowly progressive loss of kidney function leading to the need for dialysis or a kidney transplant in the 4th or 5th decade of life. Kidney biopsies from patients with FAN1 mutations exhibit markedly enlarged nuclei in renal tubular epithelial cells and interstitial fibrosis, a pathologic condition known as karyomegalic interstitial nephritis (OMIM: 614817) [3,4,5,6,7,8,9]. FAN1 patients may be more susceptible to developing cancer at extrarenal sites and sepsis after kidney transplantation [8]. While FAN1 is required for DNA interstrand crosslink repair [10,11,12,13] and countering replication stress [7,14,15], the specific target of FAN1 function in the kidney remains unknown. In addition, although exposure to environmental toxins or endogenous metabolism byproducts has been speculated to trigger KIN pathophysiology, the nature and source of the endogenous or exogenous stressor(s) responsible for causing DNA damage and on which FAN1 acts in vivo remain unclear. 

A major source of endogenous DNA damage in kidney tubular cells is the overproduction of reactive oxygen species (ROS), which can result from impaired mitochondrial oxidative metabolism, lowered antioxidant capacity, or impaired DNA repair mechanisms [16,17]. Regardless of the etiology, intracellular accumulation of ROS is associated with increased oxidative stress and oxidative DNA damage in the kidneys and is implicated in the development and progression of chronic kidney disease in humans [18,19,20,21,22]. The mitochondrial electron transport chain (ETC) is one of the major sources of endogenous ROS under normal aerobic conditions due to electron leakage at complexes I and III [19]. Mitochondria are abundant in the kidney proximal tubule cells, where they are central for fatty acid oxidation (FAO)-dependent ATP production [23]. Mitochondrial stress induced by chemicals or disease increases reactive oxygen species (ROS) generation. An increase in mitochondrial ROS negatively impacts mitochondrial energy production and can lead to nuclear DNA damage [24]. Moreover, the overproduction of ROS can increase the sensitivity of cells to other genotoxic nephrotoxins, such as mitomycin C (MMC) or cisplatin [25,26,27].

Our previous study demonstrated that FAN1-deficient kidneys have increased oxidative DNA damage after nongenotoxic obstructive injury [28], suggesting that loss of FAN1 function confers susceptibility to oxidative stress and associated DNA damage in the kidneys. This loss of FAN1 function is central to the pathophysiology of KIN in affected humans, who rarely report exposure to genotoxic injury in their clinical histories [5,8]. To test this hypothesis, we analyzed the mitochondrial function and levels of oxidative stress and DNA damage in FAN1 knockout hRTECs and FAN1-null kidneys after treatment with a subclinical dose of cisplatin that induced mitochondrial damage but had a negligible effect on nuclear DNA. Depletion of mitochondrial ROS by the mitochondrial-targeted ROS and electron scavenger JP4-039 prevented the accumulation of DNA damage in FAN1-deficient kidney tubular cells in vitro and in FAN1-null kidneys in vivo and preserved kidney function in FAN1-null mice after cisplatin injury. Our findings suggest that mitochondrial ROS are an important endogenous source of nuclear DNA damage in FAN1-deficient kidneys, which triggers the pathophysiology of karyomegalic interstitial nephritis. 

## 2. Materials and Methods

### 2.1. Mouse Lines Used and Study Approval 

FAN1KO (Ggt1-Cre dependent proximal tubule FAN1 deletion) [28] and *FAN1tm1d/tm1d* (whole body FAN1 knockout) mice that were 8–12 weeks old were backcrossed at least for 5 generations to a 129Sv-Elite background (Charles River, strain code: 476) and maintained in accordance with the IACUC requirements in the Division of Laboratory Animal Resources at the University of Pittsburgh. Animals were randomly assigned to experimental groups, which included vehicle control, cisplatin only, and cisplatin + JP4-039. Male and female mice that were 8–12 weeks old were injected intraperitoneally (i.p.) with 2 mg/kg of cisplatin (Fresenius Kabi), diluted in a normal saline solution. Control mice received normal saline injections of equivalent volumes. JP4-039 was injected by i.p. at 10 mg/kg body weight (dissolved in 50% PEG-400/50% H_2_O) starting 1 day before the cisplatin injection and continuing for 7 days after the last cisplatin administration (2-week treatment). JP4-039 (synthesized and QC’d in the laboratory of Dr. P. Wipf) [29] was prepared at a stock concentration of 0.1 mg/mL in DMSO. Mice were euthanized 14 days after the second cisplatin injection.

### 2.2. Measurement of Renal Function

Renal function was assessed with blood urea nitrogen (BUN). BUN levels in serum were measured using a kit per the manufacturer’s protocol (#K024-H1, Cayman Chemical, Ann Arbor, MI, USA).

### 2.3. Histological and Immunofluorescence Analysis of Kidney

Kidneys were fixed with 4% paraformaldehyde overnight at 4 °C, embedded in paraffin, and sectioned at 5 μm thickness. Staining with periodic acid–Schiff (PAS; #395B-1KT Sigma-Aldrich, St. Louis, MO, USA) reagents was performed according to the manufacturer’s protocols. For immunostaining, paraffin sections were rehydrated, and antigen retrieval was performed using citrate buffer (pH6). Then the sections were blocked in 10% donkey serum, 0.5% Triton, and 1% BSA in PBS for 1 h at RT and incubated with primary antibodies overnight at 4 °C. Following overnight incubation, coverslips were washed three times with PBS, incubated with a secondary antibody (Alexa Fluor, Life Technologies, Carlsbad, CA, USA) for 1 h and 30 min, rinsed with PBS, and counterstained with DAPI. Images were obtained using a Leica SP8 confocal microscope. Fluorescence was quantified using the ImageJ software (NIH, Bethesda, MD, USA).

### 2.4. Immunohistochemistry

Immunohistochemical studies were performed on sections of paraformaldehyde-fixed and paraffin-embedded kidney tissues. Endogenous peroxidases were inactivated using 3% hydrogen peroxide, followed by blocking with 10% donkey serum, 0.5% Triton, and 1% BSA in PBS. Sections were incubated with primary antibodies overnight at 4 °C. The sections were then washed and incubated for 1 h with a secondary antibody. Histochemical reactions were detected with the ABC Elite kit (Vector Laboratories, Newark, CA, USA) according to the manufacturer’s instructions. 

### 2.5. Antibodies

Primary antibodies and lectins used in this study are shown in Supplementary Table S1. Secondary antibodies included donkey anti-rabbit Alexa Fluor 594, donkey anti-rabbit Alexa Fluor 488, donkey anti-mouse Alexa Fluor 594, or donkey anti-mouse Alexa Fluor 488 (all from Molecular Probes, Eugene, OR, USA), and goat anti-rabbit IgG-HRP (sc-2004, Santa Cruz, Dallas, TX, USA). Samples were mounted in Fluoromount Aqueous Mounting Medium (F4680, Sigma-Aldrich, St. Louis, MO, USA), and images were captured on a Leica SP8 confocal microscope (Leica Microsystems, Wetzlar, Hesse, Germany). 

### 2.6. RNA Extraction and qRT–PCR 

RNA was isolated from mouse kidneys using a Quick-RNA Miniprep kit (R1055, Zymo Research, Irvine, CA, USA), and the cDNA was synthesized from 0.5 μg of total RNA using an iScript cDNA synthesis kit (Bio-Rad, Hercules, CA, USA). Quantitative real-time PCR was carried out using the SYBRGreen Master Mix and run on a CFX96 real-time PCR system (Bio-Rad, Hercules, CA, USA). Relative expression levels of mRNAs were normalized to Gapdh or 18SrRNA. Genotyping and qPCR primers used in this study are shown in Supplementary Table S2.

### 2.7. Cell Culture 

Immortalized human proximal tubule epithelial cells (hRPTECs) were cultured in DMEM/F12 medium supplemented with 5% FBS (Atlas Biologicals), 100 U/mL penicillin, 100 μg/mL streptomycin, Insulin-Transferrin-Selenium, and Glutamax-I (Gibco, Waltham, MA, USA). Cell culture plates were coated with 5 μg/cm^2^ rat tail collagen-1 (Corning 354236, Corning, NY, USA) and incubated for 1h. The cells were cultured in a humidified incubator with 5% CO_2_ at 37 °C [28]. For treatment with cisplatin and JP4-039, cells were seeded for 24 h and left untreated or treated with 5 μM cisplatin for 1 h, followed by two rises with PBS and replaced with fresh media. When JP4-039 treatment was performed following cisplatin treatment, cells were daily provided with a fresh media ±JP4-039 (5 μM) for the duration of the culture period. Treatments with the compounds were initiated when the cells were at 70% confluency.

### 2.8. YSI Metabolite Measurements

Glucose consumption and lactate production were measured using a YSI 2950 Bioanalyzer as conducted before [30]. The media was harvested 24 or 72 h later, and cells were counted to normalize for proliferation.

### 2.9. Oroboros Respirometry

Mitochondrial respiration was measured with an Oroboros Oxygraph-2K [31]. RPTEC cells were resuspended in a mitochondria isolation buffer (50 mM K-phosphate pH 7.1; 250 mM sucrose, 1 mM EDTA, 2.5% glycerol) and broken using a ball-bearing cell homogenizer (isobiotec Vertriebs UG, Heidelberg, Germany) with a 10 µM clearance. The lysate was centrifuged at 600× *g* to pellet any remaining unbroken cells and large organelles (nuclei, ER). The supernatant was centrifuged at 10,000× *g* and the resulting mitochondrial pellet was gently dispersed in MiR05 mitochondrial respiration buffer. Parental mitochondria were placed into one sealed chamber of the Oxygraph-2K and FAN1KO mitochondria into the other chamber. Cytochrome C (10 μM) was added to account for any loss of endogenous cytochrome C. Substrate additions were malate (2 mM), followed by 1.25 mM ADP to stimulate state 3 respiration. Next, 5 mM pyruvate and 5 mM glutamate were added to drive Complex I and 10 mM succinate to drive the combined activity of Complex I + II. Maximum respiration was stimulated with the uncoupler carbonyl cyanide m-chlorophenylhydrazone (CCCP; 1 μM), and finally, the Complex I inhibitor rotenone was applied to enable the determination of isolated maximal Complex II respiration. Oxygen consumption rates were normalized to protein content. The parental versus FAN1KO comparison was repeated three times with biological replicates. To facilitate the combining of data across multiple days, after protein normalization, all values within each day were further normalized to the maximum, uncoupled respiration induced by CCCP in the parental cell line mitochondria.

### 2.10. Immunofluorescence

Cells plated on coverslips were washed once with PBS and fixed with 4% paraformaldehyde for 10 min. Fixed cells were then exposed to 0.5% Triton-X 100 for 10 min for permeabilization. Cells were blocked in 10% donkey serum, 0.25% Tween, and 1% BSA in PBS for 1 h at room temperature and incubated with primary antibodies overnight at 4 °C. Following overnight incubation, coverslips were washed three times with PBS, then incubated with a secondary antibody (Alexa Fluor, Life Technologies, Carlsbad, CA, USA) for 1 h and counterstained with DAPI. Images were obtained using a fluorescence microscope. 

### 2.11. Subcellular Fractionation

Subcellular fractionations were isolated using a subcellular fractionation kit (#78840, Thermo Fisher Scientific, Waltham, MA, USA) according to the manufacturer’s instructions. The fractionated protein lysates were analyzed with western blotting using indicated antibodies. 

### 2.12. Western Blotting

Kidney tissues and hRTECs were harvested, washed with 1xPBS, and lysed in RIPA lysis buffer (Pierce, Waltham, MA, USA) supplemented with a protease and phosphate inhibitor cocktail (Pierce, #A32959, Waltham, MA, USA) and homogenized with a sonicator. Lysates were clarified by centrifugation at 16,000× *g* for 30 min at 4 °C. Gel electrophoresis of tissue lysates was performed using the NuPAGE system (Invitrogen, Waltham, MA, USA). Samples were resolved on 4–12% Bis-Tris gels and transferred onto the PVDF membrane. Membranes were blocked with 5% milk in TBST and probed with primary and secondary antibodies, respectively.

### 2.13. Measurement of Mitochondrial ROS 

Mitochondrial ROS was measured using MitoSOX Red dye (Invitrogen, M36008, Waltham, MA, USA). hRTECs were stained with 5 μM of MitoSOX Red for 30 min at 37 °C and washed 2× with PBS. MitoSOX fluorescence intensity was analyzed by flow cytometry using a BD Accuri C6 flow cytometer or treated cells and was visualized immediately with a Leica SP8 confocal microscope. Flow cytometry data were analyzed by the FlowJo software (Tree Star Inc., Ashland, OR, USA). 

### 2.14. Measurement of Cellular ROS 

Intracellular superoxide production in hRTECs was measured by fluorescence staining of CM-H2DCFDA or CellRox Green (ThermoFisher, Waltham, MA, USA) per the manufacturer’s protocol. Briefly, cells were incubated with both dyes (5 μM) for 30 min at 37 °C. After staining, the cells were washed and imaged immediately using a Leica SP8 confocal microscope. ROS generation in kidneys was assayed using CM-H2DCFDA. Unfixed kidney cryosections were incubated with 5 μM CM-H2DCFDA in PBS for 30 min at 37 °C in the dark. Samples were then washed three times, and images were obtained with a confocal microscope. The intensity of the fluorescence was quantified by the NIH Image J software.

### 2.15. ATP Measurement

ATP content in hRTECs was measured using an ATP Colorimetric Assay Kit (ab83355, Abcam, Cambridge, Great Britain) according to the manufacturer’s instructions.

### 2.16. Oil Red O Staining

For lipid staining, 4 µm frozen kidney sections were rinsed with distilled, deionized water, rinsed briefly with 60% isopropanol, stained with Oil Red O (Sigma-Aldrich, #O1391, St. Louis, MO, USA) for 1 h, and then subjected to standard hematoxylin staining and mounted on glass slides. Images were acquired under a microscope.

### 2.17. Quantification

The area occupied by Oil Red O, PPARa, and CPT1-stained sections was evaluated using ImageJ software [32]. For quantification, 6 randomly selected, non-overlapping fields were imaged at 400× magnification in the renal cortex. The values obtained were expressed as a percentage of the whole cortical area.

### 2.18. Statistical Methods

Statistical analysis was performed using GraphPad Prism 9 (GraphPad software, Boston, MA, USA). Statistical tests are two-tailed, unpaired Student’s *t*-tests or an ordinary one-way or two-way ANOVA followed by Tukey’s post hoc test for multiple group comparisons. All results are reported as means ± SEM. Significance was determined at *p* < 0.05 and represented by * to denote *p* < 0.05, ** *p* < 0.01, *** *p* < 0.001, and **** *p* < 0.0001. 

### 2.19. Data sharing Statement

RNAseq data supporting the findings of this study are openly available in the Gene Expression Omnibus repository at URL https://www.ncbi.nlm.nih.gov/geo/ (accessed on 6 July 2022), reference number GSE163862.

## 3. Results

### 3.1. KIN Is Associated with Impaired Mitochondrial Metabolism and Increased Oxidative Stress in FAN1-Deficient Kidneys

**Figure 1:**
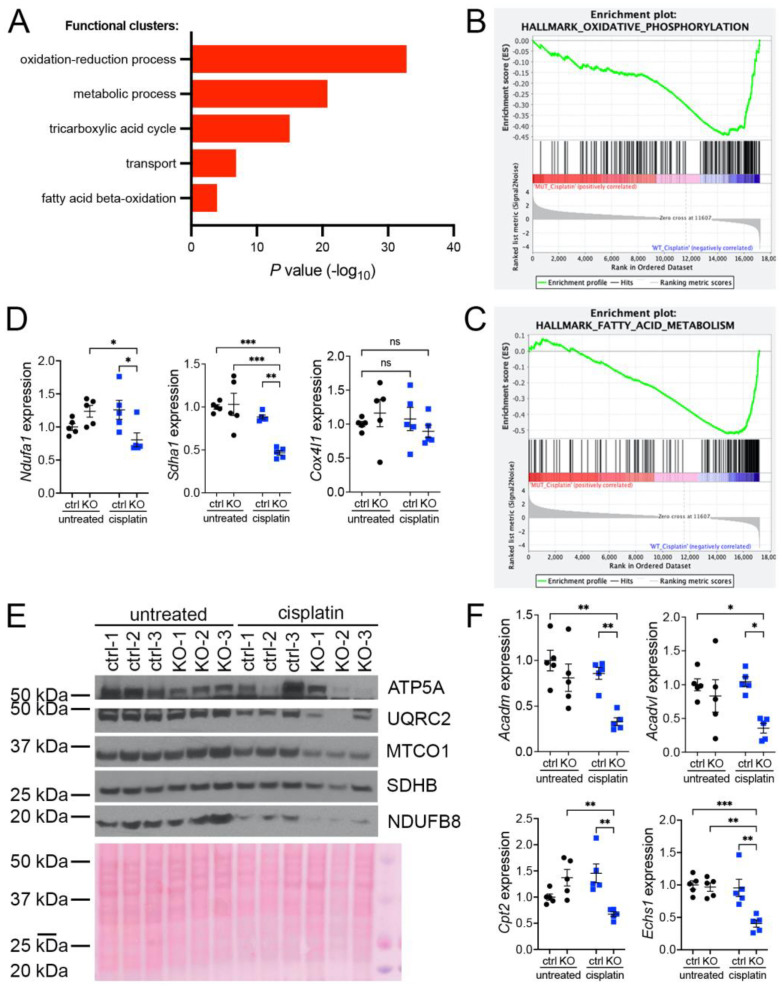
KIN is associated with impaired oxidative phosphorylation and fatty acid oxidation. (**A**) Gene ontology analysis of control and FAN1KO kidneys after repeated low-dose cisplatin injury. The graph shows −log *p* values calculated using the Benjamini–Hochberg-corrected two-tailed *t*-test for the enrichment of a specific pathway. (**B**) Gene-set enrichment signatures of oxidative phosphorylation genes in cisplatin-treated FAN1KO vs. control kidneys. Normalized Enrichment Score (NES) −2.285536, *p* < 0.0001, FDR q-value 0.0001. (**C**) Gene-set enrichment signatures of fatty acid metabolism genes in cisplatin-treated FAN1KO vs. control kidneys. Normalized Enrichment Score (NES) −2.754839, *p* < 0.0001, FDR q-value 0.0001. (**D**) qPCR analysis of *Ndufa1*, *Sdha1*, and *Cox4l1* mRNA expression. (ctrl 1.6 ± 0.3 vs. FAN1KO 6.9 ± 0.6, * *p* < 0.05, ** *p* < 0.01; *** *p* < 0.001), n = 5 each. (**E**) Western blot analysis of ATP5a, UQRC2, MTCO1, SDHB, and NDUFB8 using rodent OXPHOS mitococtail. Ponceau staining shows equal protein loading. (**F**) qPCR analysis of *Acadm*, *Acadvl*, *Cpt2*, and *Echs1* mRNA expression. (ctrl 1.6 ± 0.3 vs. FAN1KO 6.9 ± 0.6, * *p* < 0.05, ** *p* < 0.01; *** *p* < 0.001), n = 5 each. (**D**–**F**) Data are presented as the mean ± SEM. A two-way ANOVA with Tukey’s post hoc analysis.

Our previous investigations suggested that oxidative stress plays an important role in the development of KIN in FAN1-deficient mice (FAN1KO) after cisplatin injury (Supplementary Figure S1A–C and [28]). To investigate the underlying mechanism in detail, we re-examined our published RNA sequencing data by interrogating the major gene ontology groups that are significantly downregulated in FAN1KO kidneys with KIN. This analysis revealed a decrease in the expression of genes encoding components of the oxidation-reduction, metabolic, tricarboxylic acid, transport, and fatty acid beta-oxidation processes (Figure 1A). Enrichment analysis of differentially expressed genes using gene set enrichment analysis (GSEA) [33] confirmed the downregulation of genes involved in oxidative phosphorylation and fatty acid oxidation in FAN1KO kidneys during cisplatin-induced KIN pathogenesis (Figure 1B,C). Interestingly, a perturbed expression of genes in the oxidative phosphorylation and fatty acid oxidation pathways was also observed in untreated FAN1KO kidneys (Supplementary Figure S2A,B), suggesting subclinical changes in kidney homeostasis. Evaluation of the expression of several electron transport chain (ETC) components involved in oxidative phosphorylation (OXPHOS), NADH:ubiquinone oxidoreductase subunit A1 (*Ndufa1*), succinate dehydrogenase complex flavoprotein subunit A (*Sdha1*), cytochrome c oxidase subunit 4I1 (*Cox4I1*), and fatty acid oxidation (FAO), acyl-CoA dehydrogenase medium chain (*Acadm*), acyl-Coenzyme A dehydrogenase, very long chain (*Acadvl*), carnitine palmitoyltransferase 2 (*Cpt2*), enoyl Coenzyme A hydratase, short chain, 1, mitochondrial (*Echs1*), by qPCR (Figure 1D,E) or Western blot analysis and immunohistochemistry (Supplementary Figure S3) validated the RNA-seq findings in cisplatin-treated FAN1KO kidneys. 

**Figure 2:**
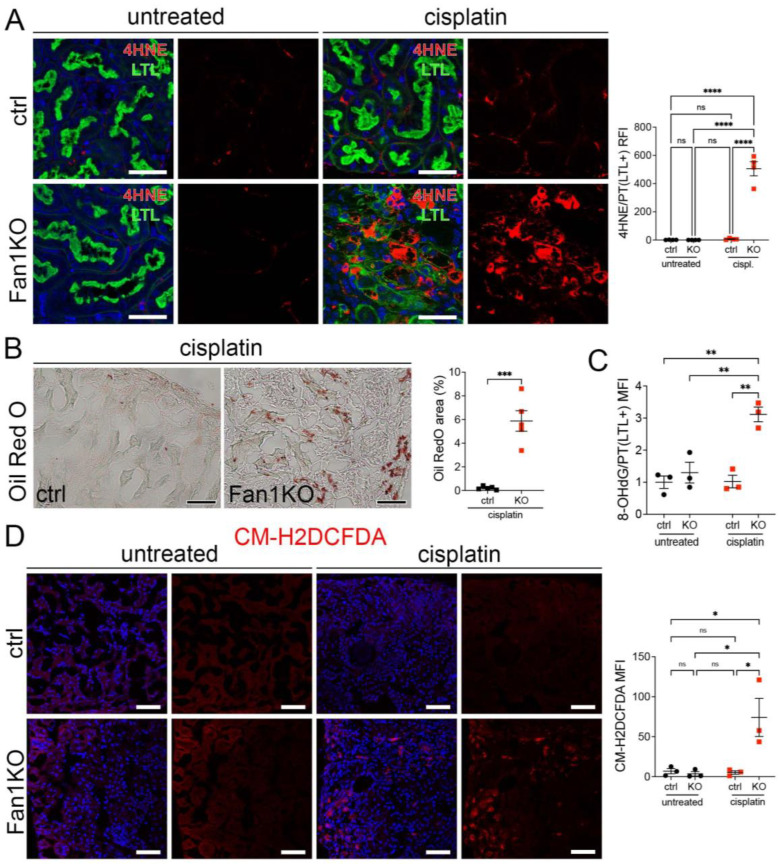
KIN pathogenesis is associated with increased lipid peroxidation, accumulation of neutral lipids, and oxidative stress in the kidney proximal tubule cells. (**A**) Representative images of cisplatin-treated control and FAN1KO kidneys stained with antibodies against 4-HNE to mark lipid peroxidation products and *Lotus tetragonolobus* lectin (LTL), which marks proximal tubule cells. **** *p* < 0.0001, n = 4 each. Scale bars: 40 μm. (**B**) Representative images of cisplatin-treated control and FAN1KO kidneys stained with OilRed O. *** *p* < 0.001, n = 4 each. Scale bars 50 μm. (**C**) Quantification of 8-OHdG staining in LTL-positive proximal tubules in untreated and cisplatin-treated wild-type and FAN1KO kidneys. ** *p* < 0.01, MFI—mean fluorescent intensity. (**D**) Quantification of CM-H2DCFDA staining in untreated and cisplatin-treated wild-type and FAN1KO kidneys. * *p* < 0.05, MFI—mean fluorescent intensity. Scale bars: 50 mm. (**A**–**D**) Data are presented as the mean ± SEM. A two-way ANOVA with Tukey’s post hoc analysis.

Furthermore, staining of FAN1KO kidneys with OilRed O, CM-H2DCFDA, or antibodies against 4-hydroxy-2-nonenal (4-HNE), which mark neutral lipids [34], cellular ROS [35], and lipid peroxidation byproducts [36], respectively, corroborated the functional defect in FAO and OXPHOS in kidneys with KIN (Figure 2A–D). In addition, the expression of key FAO regulators, peroxisome proliferator-activated receptor alpha (PPARa) and carnitine palmitoyltransferase 1A (CPT1A), was reduced in injured FAN1KO kidneys (Supplementary Figure S3A,B), demonstrating diminished energy production. Together, these data indicate that KIN pathogenesis is associated with significant deficiencies in mitochondrial energy metabolism, including in FAO and OXPHOS, which lead to increased ROS production and oxidative stress.

### 3.2. FAN1KO Proximal Tubule Cells Are Hypersensitive to Cisplatin-Induced Oxidative Stress

**Figure 3:**
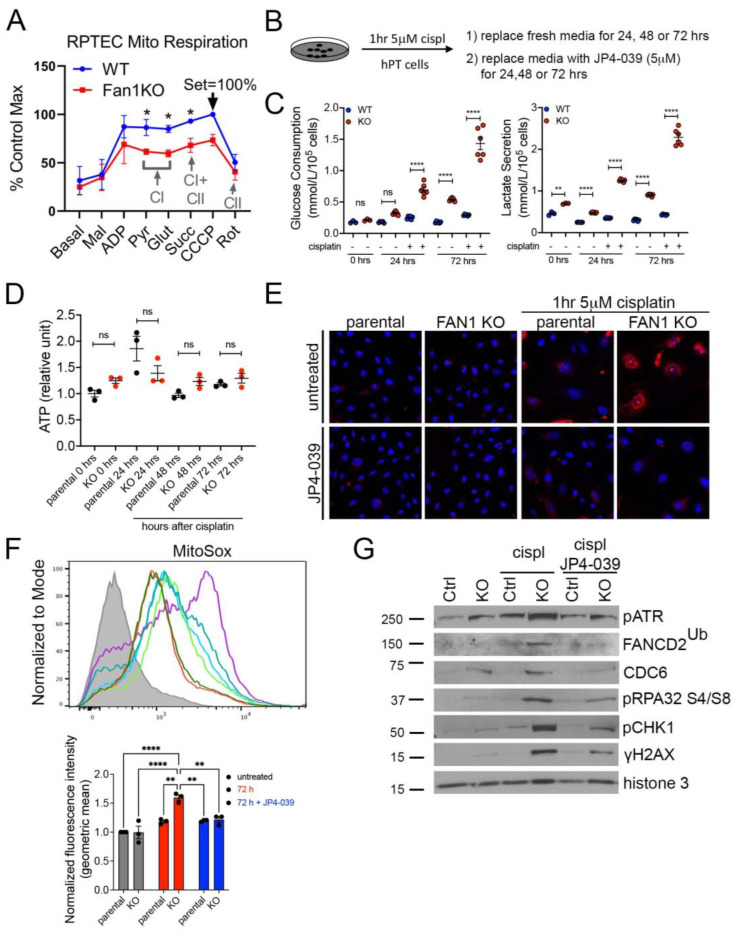
FAN1KO renal tubular epithelial cells have impaired mitochondrial metabolism and increased ROS production. (**A**) Representative oxygen consumption traces for parental (WT) and FAN1KO hRTEC mitochondria using Oroboros high-resolution respirometry. Basal, baseline respiration; CI, Electron Transport Complex I; CII, Electron Transport Complex II; Mal, malate; ADP, adenosine diphosphate; Pyr, pyruvate; Glut, glutamate; Succ, succinate; CCCP, carbonyl cyanide m-chlorophenylhydrazone; Rot, rotenone. Rotenone (CI) inhibitor was used to separate the contributions of complexes I and II. (**B**) Overview of cisplatin and JP4-039 treatment protocols in FAN1KO human renal tubular epithelial cells. Cells were seeded for 24 h and left untreated or treated with 5 μM cisplatin for 1 h, followed by two rises with PBS and replaced with fresh media. When JP4-039 treatment was performed following cisplatin treatment, cells were daily provided with fresh media ±JP4-039 (5 μM) for the duration of the culture period. (**C**) Glucose consumption and lactose secretion measurements. Parental and FAN1KO hRTECs were ± treated with cisplatin, the media were harvested, and glucose consumption and lactate production were quantified (n = 6). (**D**) ATP measurement in parental and FAN1KO hRTECs at indicated time points after treatment with cisplatin. Data are shown after normalizing ATP levels in untreated parental cells. N = 3 independent experiments. (**E**) Representative images of MitoSox staining in parental and FAN1KO hRTECs. Cells were ± treated with 5 μM cisplatin for 1 h, followed by ±JP4-039 (5 μM) for 48 h. (**F**) Detection of mitochondrial ROS (MitoSox) by flow cytometry in parental and FAN1KO hRTECs. Cells were ± treated with 5 μM cisplatin for 1 h, followed by ±JP4-039 (5 μM) for 72 h. * *p* < 0.05, ** *p* < 0.01, **** *p* < 0.0001, n = 3 independent experiments. (**G**) Representative Western blot analysis of pATR, FANCD2, CDC6, pRPA32, pCHK1, and γH2AX in FAN1KO hRTECs. Western blot analysis of DNA repair pathway markers in hRTEC chromatin preparation reveals the activation of the Fanconi anemia repair pathway (ubiquitination of FANCD2, marked by a star), increased levels of replication stress (pRPA32 S4/S8), and DNA double-strand breaks (γH2AX) in cisplatin-treated FAN1KO cells. Histone H3 is used as a chromatin loading control. Cells were ± treated with 5 μM cisplatin for 1 h followed by ±JP4-039 (5 μM) for 48 h. (**A**,**C**,**E**) Data are presented as the mean ± SEM. (**A**) A two-way ANOVA with Tukey’s post hoc analysis. (**C**,**D**) Ordinary one-way ANOVA with Tukey’s post hoc analysis.

To further examine the effect of FAN1 loss on the bioenergetic state of kidney proximal tubule cells, we measured the respiratory capacity of isolated mitochondria in human FAN1 knockout renal tubular epithelial cells (FAN1KO hRTECs) [28], using Oroboros high-resolution respirometry. Using standard culture medium, FAN1KO hRTECs showed a significantly reduced oxygen consumption compared to parental cells (Figure 3A), a defect that was localized to impaired Complex I activity as evidenced by similar rates of Complex II-driven respiration between the two cell lines upon inhibition of Complex I with rotenone. A defect in mitochondrial ETC is associated with a metabolic shift to increased glycolysis in the kidney proximal tubule cells [23,37]. To examine whether FAN1KO hRTECs are more susceptible to a genotoxin-induced glycolytic shift, we treated parental and FAN1KO cells with low-dose cisplatin and analyzed the cells at different time points (Figure 3B). Using targeted metabolite profiling, we observed significantly enhanced glucose consumption in FAN1KO hRTECs compared to parental cells, which was further augmented by treatment with cisplatin (Figure 3C). This change in glucose metabolism coincided with increased lactate secretion in FAN1KO hRTECs but not in parental cells (Figure 3C). Together, these data demonstrate that the loss of FAN1 leads to impaired mitochondrial energy metabolism and triggers glycolytic reprogramming in cultured RTECs, a phenotype that is significantly aggravated by exposure to cisplatin. Interestingly, the cellular ATP levels were not significantly different between the parental and FAN1KO hRTECs with or without cisplatin treatment (Figure 3D), suggesting that FAN1KO cells utilize glycolytic metabolism to regenerate ATP and withstand reduced OXPHOS.

The mechanism underlying cisplatin nephrotoxicity is related to its role in inhibiting mitochondrial ETC in kidney proximal tubule cells [38], which results in an increased production of mitochondrial ROS [39]. Consistent with this notion, we observed enhanced mitochondrial ROS production in FAN1KO RTECs but not in untreated or parental cells 48 h after short-term exposure to low-dose cisplatin by using MitoSox staining (Figure 3E,F), which detects mitochondrial superoxide, a specific product of OXPHOS. In contrast, when the cells were treated with the mitochondrially targeted ROS and electron scavenger JP4-039 [40,41,42] immediately after exposure to cisplatin, we observed a significant reduction in the accumulation of mitochondrial ROS in FAN1KO hRTECs (Figure 3E,F), further demonstrating that FAN1 inactivation leads to increased susceptibility to mitochondrial functional impairment in RTECs. Consistent with the increase in mitochondrial ROS, cisplatin-treated FAN1KO cells displayed a significant upregulation in total cellular ROS, as measured by CM-H2DCFDA and CellROX staining (Supplementary Figure S4A,B). Again, treatment with JP4-039 prevented the upregulation in total ROS production, consistent with the notion that their generation was secondary to mitochondrial ROS. 

We have previously shown that the loss of FAN1 sensitizes human kidney proximal tubule cells to cisplatin-induced DNA damage, which causes replication stress and activates chronic DNA damage response signaling [28]. Cisplatin’s nephrotoxicity, apart from its effect on mitochondrial ETC and ROS production, is also caused by the formation of DNA adducts in the nucleus [43]. Incomplete repair of DNA oxidative damage or DNA adducts causes DNA double-strand and single-strand breaks and leads to persistent activation of ATM and ATR kinases, respectively. Indeed, we observed the activation of ATM and ATR in FAN1KO hRTECs after treatment with cisplatin (Supplementary Figure S5). To investigate the differential contribution of cisplatin-induced oxygen toxicity (ROS) vs. DNA adducts in FAN1 defective pathogenesis, we treated parental and FAN1KO hRTECs with cisplatin +/− JP4-039 (5 μM in DMSO) and analyzed the expression of different DNA damage markers and replication factors in the chromatin fraction of hRTECs 48 h later by Western blotting (Figure 3G). Treatment with JP4-039 after short-term exposure to cisplatin significantly reduced replication stress and DNA damage in FAN1KO cells, demonstrating that ROS and oxidative damage play an important role in the cellular pathophysiology of FAN1-deficient kidney tubular epithelial cells. 

### 3.3. Treatment with Mitochondrial ROS Scavenger JP4-039 Reduces Tubular Damage and Prevents KIN Pathogenesis in FAN1-Null Mice

**Figure 4:**
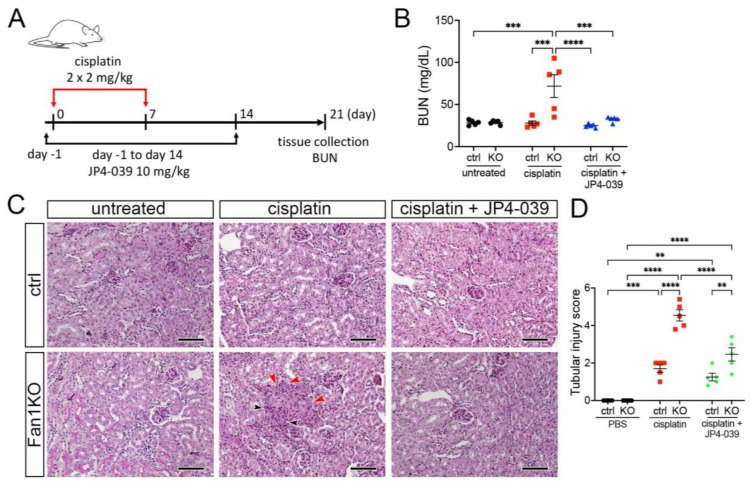
DNA damage causes aberrant cell cycle activity in FAN1-null kidneys. (**A**) Mice received 2 doses of intraperitoneal (i.p.) cisplatin (2 mg/kg) vs. vehicle (normal saline) on days 0 and 7 and were treated with i.p. JP4-039 (10 mg/kg in 50% PEG-400/50% H_2_O) vs. vehicle (50% PEG-400/50% H_2_O) 1 day prior to and until day 14 after the first cisplatin injection. Tissues were collected for analysis 21 days after the first cisplatin dose. (**B**) Blood urea nitrogen (BUN) measurements in ctrl and FAN1-null mice show loss of kidney function in FAN1KO mice after induction of KIN. *** *p* < 0.001, **** *p* < 0.0001, n = 5 each. (**C**) Histological analysis of kidney sections by Periodic acid–Schiff (PAS) staining demonstrates the formation of KIN in FAN1KO mice, characterized by tubular atrophy, formation of karyomegalic nuclei (red arrowheads), and segmental basement membrane thickening (black arrowheads) in the proximal tubules. Scale bars: 50 μm. (**D**) Tubular injury scores in control mice were compared with those in FAN1KO kidneys after low-dose cisplatin administration. ** *p* < 0.01, *** *p* < 0.001, **** *p* < 0.0001, n = 5 each. (**B**,**D**) Data are presented as the mean ± SEM. A two-way ANOVA with Tukey’s post hoc analysis.

To extend the in vitro findings and evaluate whether suppression of mitochondrial ROS can be used to suppress KIN pathogenesis in vivo, we generated a whole-body FAN1 deletion mouse strain (FAN1-null) and treated the mice biweekly with a low dose of cisplatin (2 mg/kg) combined with the daily administration of 10 mg/kg JP4-039 or vehicle control (Figure 4A). This cisplatin dosing regimen was sufficient to induce kidney injury in FAN1-null animals but not in wild-type mice (Figure 4B,C). Importantly, administration of JP4-039 for the first two weeks after the initiation of cisplatin treatment attenuated cisplatin-induced renal failure in FAN1-null mice (Figure 4B). The renoprotective effects of JP4-039 were further demonstrated by the preservation of kidney histology and reduced tubular injury in PAS-stained FAN1-null kidney sections compared with kidneys from cisplatin-treated mice (Figure 4B). Consistent with the histology-based analysis, the immunofluorescence staining of kidney injury molecule-1 (KIM1) was significantly diminished in cisplatin-treated FAN1-null kidneys after JP4-039 administration (Supplementary Figure S6A,B), indicating reduced tubular injury. 

**Figure 5:**
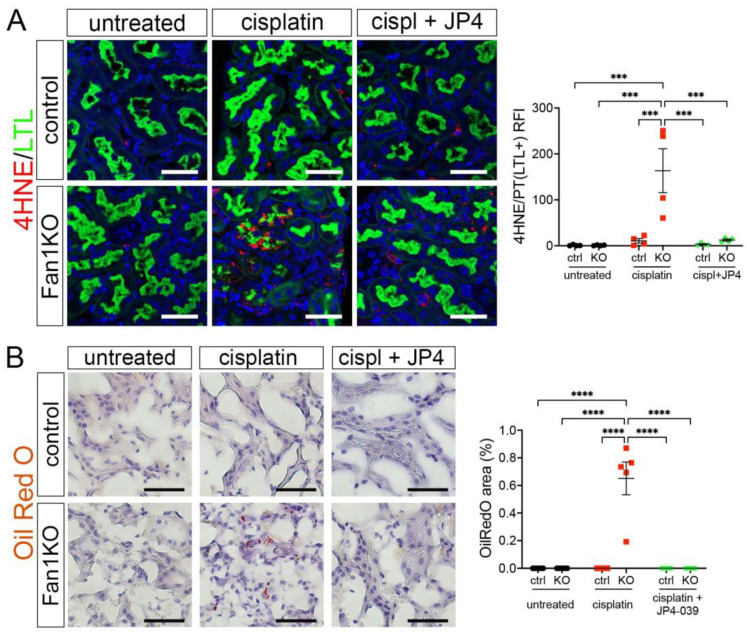
Oxidation stress-induced tubular damage is attenuated by JP4-039. (**A**) Representative images of untreated and cisplatin (2 × 2 mg/kg) ±JP4-039 (10 mg/kg in 50% PEG-400/50% H_2_O) treated control and FAN1KO kidneys stained with antibodies against 4-HNE to mark lipid peroxidation products. *** *p* < 0.001, n = 4 each. Scale bar: 25 μm. (**B**) Increased lipotoxicity in injured FAN1KO kidneys as revealed by OilRed O staining. Treatment with JP4-039 reduces lipid accumulation in cisplatin-injured FAN1KO kidneys. **** *p* < 0.0001, n = 5 each. Scale bar: 50 μm. (**A**,**B**) Data are presented as the mean ± SEM. A two-way ANOVA with Tukey’s post hoc analysis.

Because our in vitro studies had suggested that the loss of FAN1 pathogenesis is driven by increased ROS levels in the kidney proximal tubular cells, we next examined the expression of oxidative stress markers in FAN1-null kidneys. As seen in Figure 5A, cisplatin led to a strong upregulation of the lipid peroxidation marker 4-HNE in FAN1-null kidneys, which was abolished by the JP4-039 administration. Similarly, JP4-039 suppressed the levels of total cellular ROS (CM-H2DCFDA) (Supplementary Figure S7A) and prevented the expression of the DNA damage marker γH2AX (Supplementary Figure S7B) and DNA oxidation marker 8-OHdG (Supplementary Figure S7C) in cisplatin-treated FAN1-null kidneys. In contrast, all of these oxidative stress markers were strongly upregulated in cisplatin-treated FAN1-null kidneys but not in cisplatin-treated control kidneys, demonstrating that increased mitochondrial ROS production in the absence of FAN1 DNA repair activity plays a causal role in the tubular pathogenesis of KIN. 

Our earlier analysis had shown that KIN pathogenesis is associated with impaired FAO (Figure 1). Therefore, we examined whether JP4-039 mediated reduction in cisplatin-induced mitochondrial ROS production also improves mitochondrial fatty acid oxidation and prevents intracellular lipid deposition in cisplatin-injured FAN1-null kidneys. Indeed, the formation of lipid droplets was suppressed in JP4-039 treated mice compared with cisplatin-treated mice (Figure 5B), suggesting improved lipid metabolism. This notion was supported by the observation that treatment with JP4-039 increased the protein expression levels of PPARα and CPT1 in cisplatin-treated FAN1-null kidneys compared to cisplatin-injured FAN1-null mice (Supplementary Figure S8A,B). These data demonstrate that impaired mitochondrial FAO contributes to tubular pathogenesis in FAN1-deficient kidneys. Taken together, these in vivo results in FAN1-null mice corroborate and extend our previous findings [28], demonstrating that unhindered accumulation of cellular ROS and increased oxidative damage to lipids and nuclear DNA underpin tubular cell injury and the progression of KIN pathogenesis in FAN1-deficient kidneys.

## 4. Discussion

While the presence of bi-allelic pathogenic variants of the FAN1 gene has been shown to cause the DNA repair deficiency syndrome KIN in humans and mice [1,28,44,45], the endogenous molecular events that can drive KIN pathogenesis in the kidney tubular epithelial cells have remained enigmatic. Most of the earlier studies investigating the molecular causes of KIN have focused on studying FAN1 function at the level of DNA replication and repair [7,28,44]. In this study, we show that FAN1 function in DNA repair is linked to mitochondrial energy metabolism in the kidney tubular epithelial cells through increased sensitivity of FAN1-deficient kidneys to mitochondrially derived oxygen stress. 

To investigate the role of FAN1 in mitochondrial metabolism, we employed the chronic cisplatin kidney injury model since our earlier work had demonstrated that cisplatin treatment accelerates KIN pathogenesis in FAN1-deficient kidneys [2,28], presumably due to inefficient repair of DNA interstrand crosslinks (ICL), which are the biological target of FAN1 function [10,11,12,46]. However, by reanalyzing our previously published genome-wide transcriptomics data from whole kidney samples of proximal tubule-specific FAN1-deficient (FAN1KO) mice [28], we made the surprising discovery that mitochondrial energy metabolism pathways related to oxidative phosphorylation (OXPHOS) and fatty acid oxidation (FAO) were perturbed at baseline in FAN1KO kidneys without prior exposure to cisplatin, compared to wild-type kidneys. This suggested that FAN1KO kidneys are hypersensitive to an endogenously produced physiological stressor, which can trigger subclinical pathological changes related to KIN in unchallenged FAN1KO mice. Alterations in the expression of OXPHOS and FAO pathway genes were further amplified after cisplatin treatment in Fan1KO kidneys, leading to accelerated KIN pathogenesis but not in wild-type kidneys, further demonstrating a functional connection between FAN1, mitochondrial energy metabolism, and KIN pathophysiology in the kidney tubular epithelial cells. Our phenotypic analysis, which included both male and female animals in all experimental cohorts, did not reveal significant gender-specific differences in response to low-dose cisplatin injury, suggesting that FAN1 kidney pathophysiology is not gender-dependent in mice.

Functionally, demonstrating that FAN1 hRTECs have reduced OXPHOS activity and enhanced glycolytic metabolism under normal cell culture conditions provides compelling evidence that FAN1 cells have a deficient energy metabolism due to impaired Complex I activity. Mitochondrial Complex I accepts reducing equivalents from NADH, thereby driving ATP production as well as regenerating NAD+, a crucial metabolic cofactor for many cellular processes. Because NADH produced during glycolysis in the cytosol can also be regenerated by Complex I through the glycerol phosphate shuttle, impaired Complex I may also contribute to the elevated glycolysis and production of lactate in FAN1-deficient cells. In addition, the diminished expression of *Ndufa1* (Complex I) and *Sdha* (Complex II) genes was observed in cisplatin-treated FAN1KO kidneys, mirroring the functional defect observed in FAN1KO cells. Further, a shift to glycolytic metabolism in cisplatin-treated FAN1KO kidneys was suggested by a significant downregulation in FAO genes. While the individual gene expression of OXPHOS or FAO components, whose expression we measured with qPCR, was not significantly changed in untreated FAN1KO kidneys, we observed a differential enrichment in the expression of OXPHOS and FAO GSEA gene sets in FAN1KO kidneys compared with wild-type kidneys, suggesting functional perturbations at a pathway level. This notion is supported by the clear downward trend in the expression of *Acadm* and *Acadvl* in untreated FAN1KO kidneys, indicative of impaired FAO, the main source of energy for kidney proximal tubule cells [23]. Functionally, a reduction in FAO gene expression was associated with intracellular lipid accumulation in FAN1KO and FAN1-null kidneys, which has been demonstrated to contribute to the development of tubular atrophy and kidney fibrosis [47,48]. These data demonstrate that a decline in mitochondrial FAO is among the mechanisms underlying KIN in FAN1-deficient kidneys.

Reduction in mitochondrial OXPHOS activity leads to increased mitochondrial ROS overproduction and oxidative stress in kidney tubular cells [38,49] and has been associated with the development of chronic kidney disease in various mouse models of kidney injury [50,51]. Elevated ROS levels and oxidative DNA damage (8-OHdG) were reported in humans with acute kidney injury and chronic kidney disease [21,52,53]. We found that FAN1KO hRTECs exhibited a decline in oxidative phosphorylation and increased glycolysis when cultured under normal cell culture conditions at atmospheric oxygen levels, suggesting that FAN1 cells have enhanced sensitivity to oxidative stress. Indeed, exposure of FAN1 knockout RTECs or FAN1-null mice to subclinical levels of cisplatin-induced ROS overproduction and oxidative DNA damage, which were presented in both models by treatment with the mitochondrially targeted antioxidant JP4-039, demonstrates that ROS are critical for triggering the KIN pathophysiology. Intriguingly, analogous hypersensitivity to oxidative stress has been previously demonstrated in animal and cell culture models defective in Fanconi anemia [26,54,55,56,57] or excision repair cross complementation group 1 (ERCC1) [58] DNA repair pathway genes, conditions that were improved by antioxidant treatment [58,59].

This is the first study that suggests a mechanistic link between oxidative stress, DNA damage, and KIN pathogenesis. It is noteworthy that most of the individuals diagnosed with KIN have no reported exposure to chemotherapeutics or nephrotoxic medicines in their clinical history [5,8,60]. Based on our findings, we propose that FAN1/KIN kidney pathogenesis in humans is triggered by physiological levels of ROS, which cause persistent oxidative DNA damage in the kidney proximal tubule cells due to impaired DNA repair mechanisms. Chronic DNA damage is energetically stressful to the cell and leads to a subclinical decline in mitochondrial function and a further increase in cellular ROS production and oxidative stress, which ultimately manifests in clinical tubular injury and interstitial fibrosis in patients with KIN [3,28,44,45]. Mitochondrial abnormalities, accumulation of DNA damage, and tubular injury were significantly improved by JP4-039-mediated ROS scavenging, suggesting that FAN1/KIN kidney pathology, which currently has no specific treatment, may benefit from targeted antioxidant therapy. Although our work shows that ROS is a relevant source of endogenous DNA damage in FAN1-deficient kidneys, other endogenous stressors, such as formaldehyde [61] or spontaneous genomic instability, can potentially also trigger KIN pathophysiology. Furthermore, patients with biallelic FAN1 pathogenic variants may also be at increased risk of cancer and acute kidney injury from treatment with cisplatin or other environmental toxins.

## 5. Conclusions

Taken together, the data presented here show for the first time that redox imbalance and oxidative stress are important hallmarks of FAN1/KIN pathophysiology in the kidney and suggest that mitochondrial ROS is a novel target for mitigating KIN pathogenesis. 

## Data Availability

Data is contained within the article or supplementary material. RNA-seq data is available in the Gene Expression Omnibus repository at URL https://www.ncbi.nlm.nih.gov/geo/ (accessed on 27 February 2023), reference number GSE163862.

## Supplementary Materials

The following supporting information can be downloaded at: https://www.mdpi.com/article/10.3390/antiox12040900/s1, Figure S1: Repeated low-dose cisplatin administration triggers KIN pathogenesis in FAN1KO kidneys; Figure S2: KIN is associated with impaired oxidative phosphorylation and fatty acid oxidation; Figure S3: Chronic cisplatin injury leads to reduced PPARa and CPT1 expression in FAN1KO kidneys; Figure S4: JP4-039 blocks the formation of cellular ROS in cisplatin-treated FAN1KO hRTECs; Figure S5: FAN1KO hRTECs are hypersensitive to cisplatin toxicity; Figure S6: JP4-039 prevents tubular injury in cisplatin-treated FAN1-null kidneys; Figure S7: JP4-039 suppresses oxidative stress and DNA damage in cisplatin-treated FAN1-null kidneys; Figure S8: JP4-039 prevents the loss of FAO enzymes in cisplatin-treated FAN1-null kidneys; Table S1: List of antibodies and lectins used in the study; Table S2: List of genotyping and quantitative RT-PCR primers and NCBI gene accession numbers.
