# Supplementary material for: Mitochondrial ROS Triggers KIN Pathogenesis in FAN1-Deficient Kidneys
Source: Antioxidants (Basel). 2023 Apr 8;12(4):900. doi: 10.3390/antiox12040900 (PMC10135478; doi:10.3390/antiox12040900)
Supplement: Supplementary file 1 [file antioxidants-12-00900-s001.zip › antioxidants-2282843-supplementary.pdf]

SUPPLEMENTARY FIGURES AND TABLES

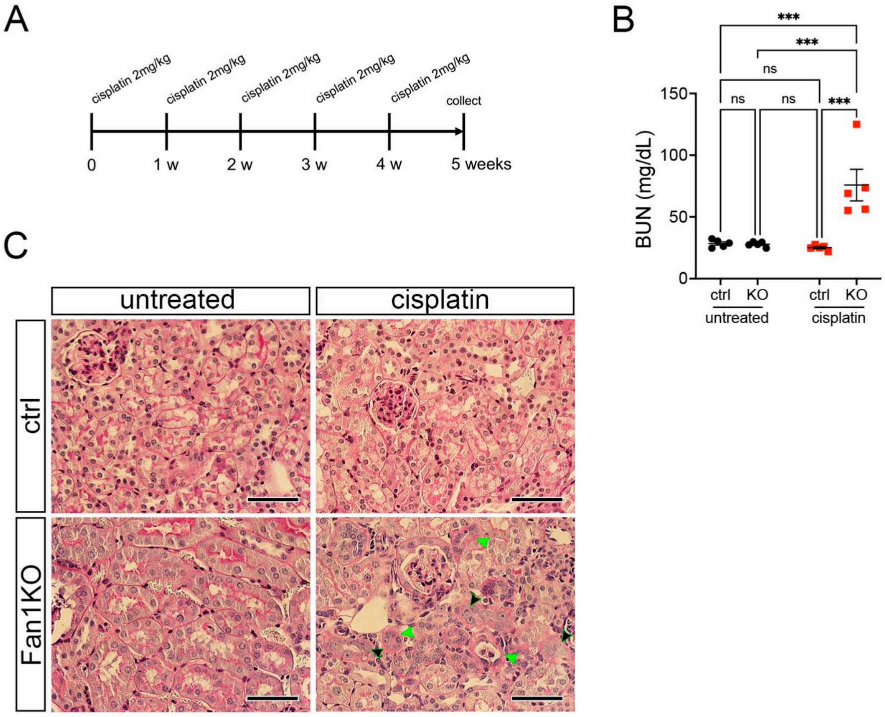

Supplementary Figure S1. Repeated low dose cisplatin administration triggers KIN pathogenesis in *Fan1* KO kidneys.

(**A**) Schematic diagram of repeated low dose cisplatin administration.

(**B**) Blood urea nitrogen levels were measured at the time of kidney collection, 5 weeks after the first cisplatin injection. 2-way ANOVA, n=5 mice each group, ***$p<0.001$.

(**C**) Representative images of Periodic acid–Schiff (PAS) stained kidney samples. *Fan1* KO mice develop KIN after 5 weeks of cisplatin injection, characterized by tubular atrophy, formation of karyomegalic nuclei (black arrowheads) and segmental basement membrane thickening (green arrowheads) in the proximal tubules. Scale bars 60 μm.

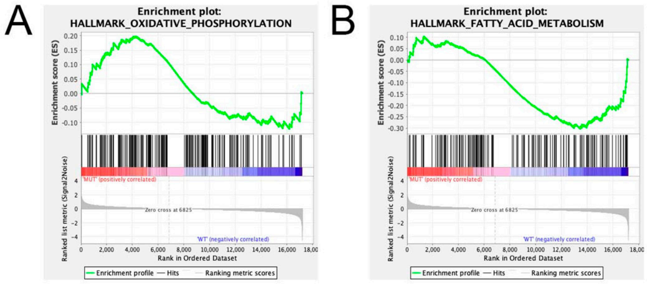

Supplementary Figure S2. KIN is associated with impaired oxidative phosphorylation and fatty acid oxidation.

(**A**) Gene-set enrichment signature of oxidative phosphorylation (OXPHOS) genes in untreated Fan1KO vs control kidneys. OXPHOS gene set is upregulated in *Fan1* KO kidneys.

(**B**) Gene-set enrichment signature of fatty acid metabolism (FAO) genes in untreated Fan1KO vs control kidneys. FAO gene set is down regulated in *Fan1* KO kidneys.

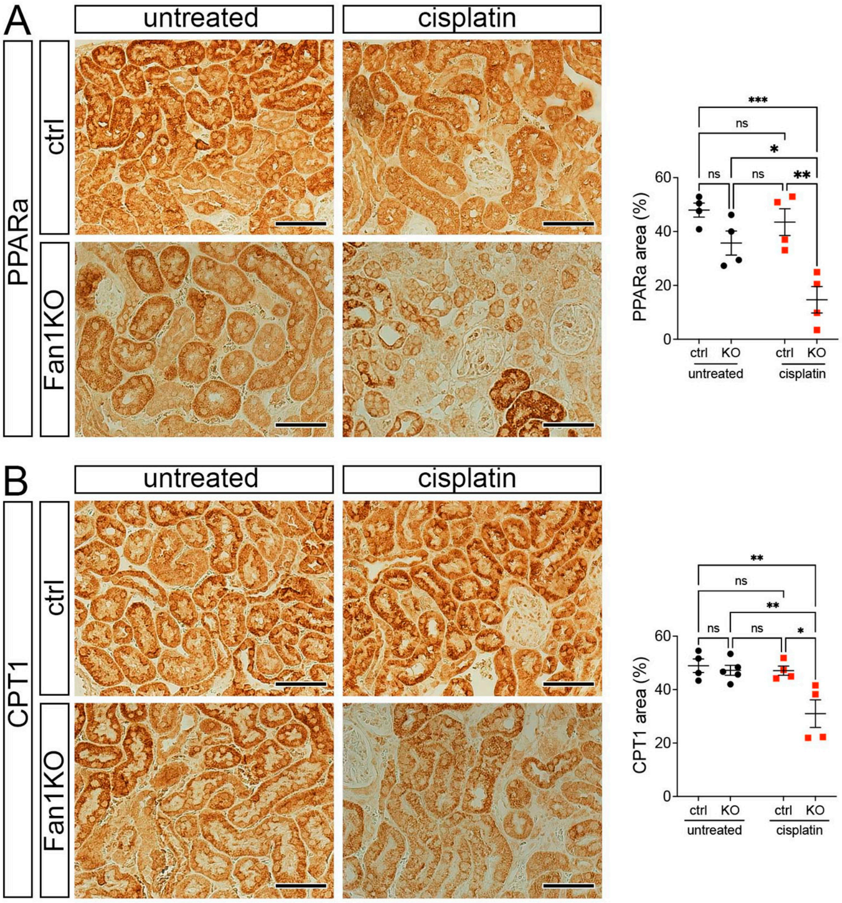

Supplementary Figure S3. Chronic cisplatin injury leads to reduced PPARa and CPT1 expression in Fan1 KO kidneys.

(**A**) Representative immunohistochemical staining of PPARa in control and *Fan1* KO kidney samples +/- treated with cisplatin. Scale bars 60 μm. Quantification of PPARa immunostaining in randomly chosen fields of kidney cortical areas. *p<0.05, **p<0.01, ***p<0.001, n=4 each.

(**B**) Representative immunohistochemical staining of CPT1 in control and *Fan1* KO kidney samples +/- treated with cisplatin. Scale bars 60 μm. Quantification of CPT1 immunostaining in randomly chosen fields of kidney cortical areas. *p<0.05, **p<0.01, n=4 each. (**A-B**) Data are presented as the mean ± SEM. A 2-way ANOVA with Tukeys' post hoc analysis.

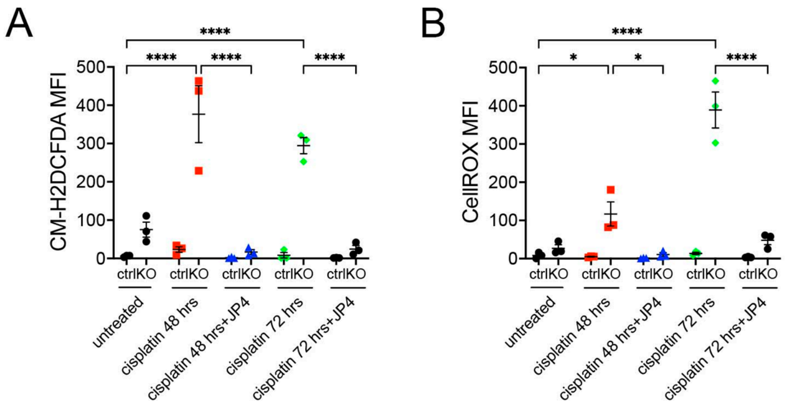

Supplementary Figure S4. JP4-039 blocks the formation of cellular ROS in cisplatin treated FAN1KO hRTECs.

(**A**) Quantification of CM-H2DCFDA staining in untreated and cisplatin ± JP4-039 treated parental and FAN1KO hRTECs. ****$p<0.0001$, n=4 each. MFI – mean fluorescence intensity (arbitrary units).

(**B**) Quantification of CellRox staining in untreated and cisplatin ± JP4-039 treated parental and FAN1KO hRTECs. *$p<0.05$, ****$p<0.0001$, n=4 each. MFI – mean fluorescence intensity (arbitrary units). (**A**-**B**) Data are presented as the mean ± SEM. A 2-way ANOVA with Tukeys' post hoc analysis.

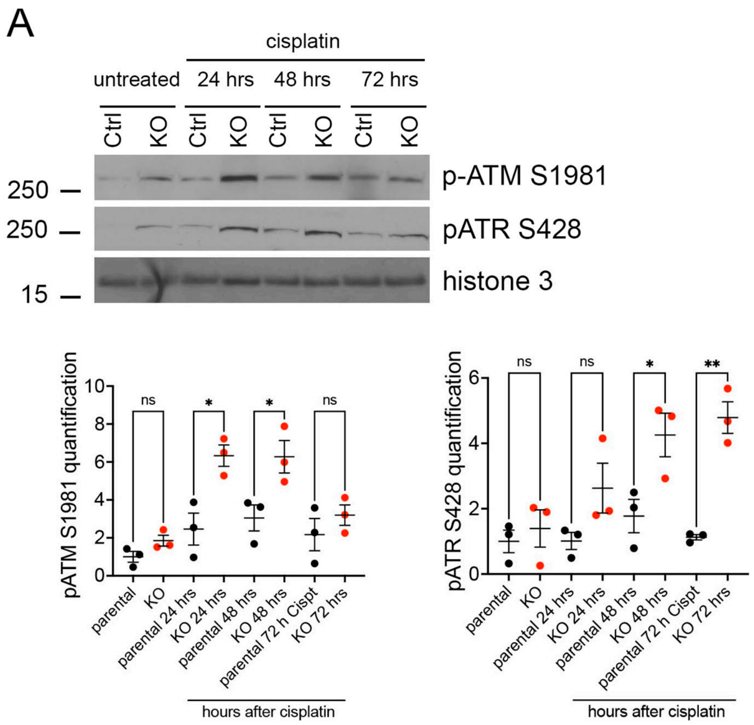

Supplementary Figure S5. FAN1KO hRTECs are hypersensitive to cisplatin toxicity.

(**A**) Representative Western blot analysis of pATM-S1981 and pATR-S428 expression in parental and FAN1KO hRTECs. Histone H3 is used as a chromatin loading control. Cells were ± treated with 5 µM cisplatin for 1 hour and collected for analysis 24 hours, 48 hours and 72 hours later. Quantification of pATM-S1981 and pATR-S428 levels in parental and FAN1KO hRTECs is based on 3 independent experiments. *$p<0.05$, **$p<0.01$ n=3 each. Data are presented as the mean ± SEM. Ordinary 1-way ANOVA with Tukeys' post hoc analysis.

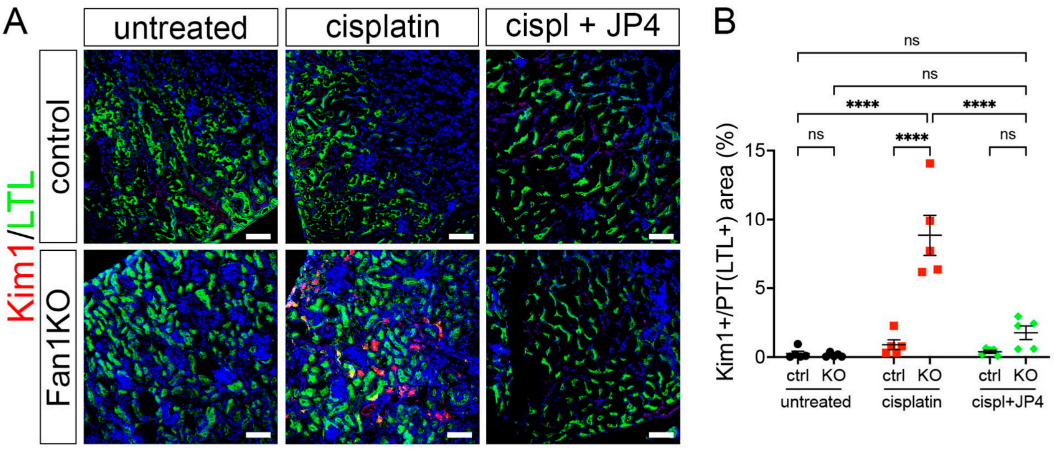

Supplementary Figure S6. JP4-039 prevents tubular injury in cisplatin treated *Fan1*-null kidneys.

(**A**) Kim1 expression analysis by immunofluorescence staining in untreated and cisplatin ± JP4-039 treated control and Fan1KO kidneys. Scale bar 75 µm.

(**B**) Quantification of the Kim1-positive area in LTL-positive proximal tubules shows a significant upregulation of Kim1 in Fan1KO kidneys. ****p<0.0001, n=5 each. Data are presented as the mean ± SEM. A 2-way ANOVA with Tukeys' post hoc analysis.

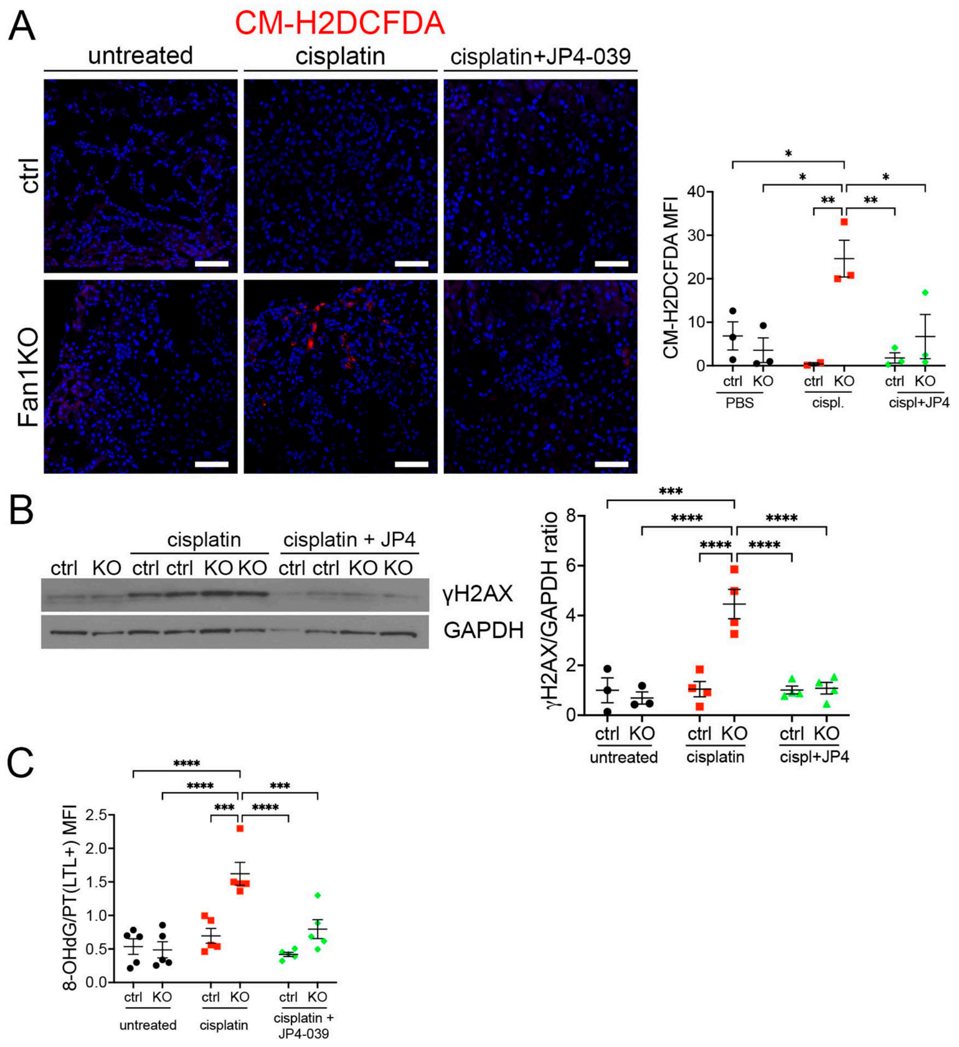

Supplementary Figure S7. JP4-039 suppresses oxidative stress and DNA damage in cisplatin treated *Fan1*-null kidneys.

(**A**) Quantification of CM-H2DCFDA staining in untreated and cisplatin ± JP4-039 treated kidneys. *$p<0.05$, **$p<0.01$. MFI - mean fluorescent intensity. Scale bar 50 μm.

(**B**) Western blot analysis of γH2AX in untreated and cisplatin ± JP4-039 treated kidneys. GAPDH is a loading control. Densitometric analysis was performed using NIH ImageJ software (n=3-4). ***$p<0.001$, ****$p<0.0001$.

(**C**) Quantification of 8-OHdG staining in LTL-positive proximal tubules in untreated and cisplatin ± JP4-039 treated kidneys. ***$p<0.001$, ****$p<0.0001$. 2way ANOVA, MFI - mean fluorescent intensity.

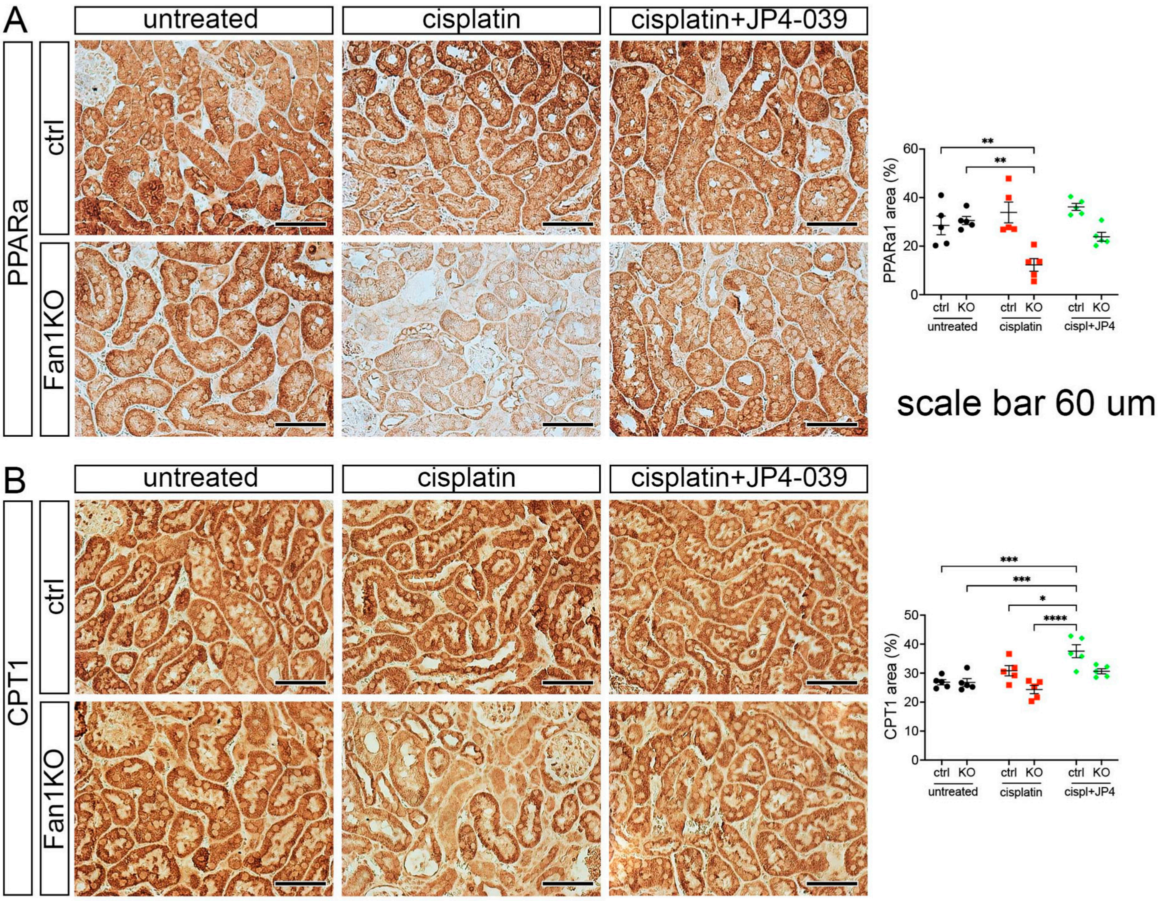

Supplementary Figure S8. JP4-039 prevents the loss of FAO enzymes in cisplatin treated *Fan1*-null kidneys.

(**A**) Immunohistochemistry staining for PPARa in the kidneys of mice. Scale bars 60 μm. Data are presented as mean ± SEM. **p<0.01.

(**B**) Immunohistochemistry staining for carnitine palmitoyltransferase-1a (CPT1a) in the kidneys of mice. Scale bars 60 μm. Data are presented as mean ± SEM. *p<0.05, ***p<0.001, ****p<0.0001.

Supplementary Table S1. Antibodies and lectins used in this study

Antibody	Source	Identifier
Rodent OxPhos	ThermoFisher	#45-8099
Rabbit Phospho-Histone H2A.X (Ser139)	Cell Signaling	#9718
Rabbit 4-Hydroxynonenal (4-HNE)	R&D systems	MAB3249
Rabbit PPARA	Proteintech	15540-1-AP
Rabbit CPT1A	Proteintech	15184-1-AP
Phospho-ATM (Ser1981)	Cell Signaling	#5161
Phospho-ATR (Ser428)	Cell Signaling	#2853
Rabbit FANCD2	Abcam	ab108928
Mouse GAPDH	Santa Cruz	sc-47724
Rat TIM-1/KIM-1/HAVCR	R&D systems	MAB1817
Rabbit Phospho-RPA32 (S4/S8)	Bethyl Laboratories	A300-245A
Rabbit Histone H3	Cell Signaling	#9715
LTL lectin	VectorLaboratories	FL-1321
Mouse CDC6	Santa Cruz	sc-9964
Rabbit Phospho-Chk1	Cell Signaling	#2348
Mouse 8-oxo-dG	R&D systems	4354-MC-050
Rabbit Histone H3	Cell Signaling	#9715

Supplementary Table S2. qRT-PCR primers and NCBI gene accession numbers

Gene	Species	Forward	Reverse	NCBI
Acadm	mouse	AACACAACACTCGAAAGCGG	TTCTGCTGTTCCGTCAACTCA	NM_007382
Acadvl	mouse	CTACTGTGCTTCAGGGACAAC	CAAAGGACTTCGATTCTGCCC	NM_017366
CoxIV	mouse	ATATTAACCGGCGCTACGAC	ATAGTCCCACTTGGCGGAGA	NM_001293559
Cpt2	mouse	CACAACATCCTGTCCACCAG	GCATCGAACATGTCTTCCAA	NM_009949
Echs1	mouse	AGCCTGTAGCTCACTGTTGTC	ATGTACTGAAAGTTAGCACCCG	NM_053119
Gapdh	mouse	GACTTCAACAGCAACTCCCA	TGTAGCCGTATTCATTGTCATACC	NM_001289726
Ndufa1	mouse	ATTATGGGGGTGTGCTTGGT	ATCGCGTTCCATCAGATACC	NM_019443
Sdha1	mouse	GGAACACTCCAAAAACAGACCT	CCACCACTGGGTATTGAGTAGAA	NM_023281
